# Around the Hospitals

**Published:** 1892-07-23

**Authors:** 


### Around the Hospitals.

**The Derbyshire Hospital for Sick Women** has now been in working order for rather more than a year. Since the first patient was admitted in May, 1891, 34 in-patients have been received, and many out-patients have attended. The institution is much appreciated already, a fact shown by the desire expressed by patients to hold a sale of work contributed by themselves for the benefit of the hospital. Some alterations in the buildings were already in progress, including a specially-designed operating theatre.

**The Chesterfield and North Derbyshire Hospital** reports a large gratifying increase in the subscriptions to the institution by the working classes during the past year. The hospital has received one or two legacies, but otherwise the receipts for the year have not been quite so large as usual. In the death of the Duke of Devonshire the institution lost a much-valued President. The erection of the new wing, which has been for some time in progress, was much delayed owing to difficulties arising in the matter of the foundation. To cover the cost of the additional buildings subscriptions will be appealed for later on.

**The London Lock Hospital**, Harrow Road, which is the only one of its kind in the Metropolis, is deserving of a large amount of support, fulfilling as it does the twofold object of rescue in cases of physical and moral necessity. Patients come from all parts of the kingdom, and the special appeal for £5,000, very urgently needed, should meet with adequate response beyond the limits of the Metropolis. During the last six years 5,300 patients have been admitted, and of these it is stated 1,400 are doing well and earning their own livelihood. The immense advantage of the industrial instruction to the inmates which follows the hospital treatment cannot be over-estimated, and the institution should receive every encouragement from the public. The period spent in the institution is generally one year, but the inmates frequently ask to remain longer. At the end of a year's residence an outfit is provided and a situation found. A gratuity is added of one guinea where a good character is earned after twelve months' service. Thus every encouragement and assistance is afforded, even after inmates have left the hospital, making the work as thorough as it is possible to be.

**The Hospital for Women and Children**, Cork, has had a verdict of independence forced upon it this year, and must henceforth carry on its existence unaided by the helping hand from some larger institution. There has been a strong feeling of late in Cork that the medical charities would benefit by amalgamation, and, in accordance with this view, the Committee of the Women's and Children's Hospital proposed to unite it to the South Infirmary. The South Infirmary having, however, declined, the proposal has been abandoned. There are some very encouraging features in the record of the past year's work of the institution. Two ladies have each endowed a cot, and have undertaken to support another, whilst the matron, Miss Baxter, a most energetic worker for the hospital, has undertaken to defray out of her special fund the cost of the new bath-room wing, which has been lately completed. The expenses of painting and decorating during the past year have been paid from the same source. The hospital, which is the only one of its kind in the south of Ireland, contains 62 beds. Great economy, and management must be exercised, as the cost of maintenance per bed amounts to only £30 1s. 8½d. We notice in the accounts as shown in the annual report that provisions have decreased by £129 during the year, which, taking the fact into consideration that there were eighteen fewer patients, is still rather a large diminution. The report issued by the institution is an admirable one, and testifies to the capability and personal interest displayed by the management.

